# Effect of Hcp Iron Ion Regulation on the Interaction Between *Acinetobacter baumannii* With Human Pulmonary Alveolar Epithelial Cells and Biofilm Formation

**DOI:** 10.3389/fcimb.2022.761604

**Published:** 2022-02-23

**Authors:** Ping Pan, Xiaolei Wang, Yi Chen, Qiong Chen, Yunxing Yang, Chenxing Wei, Tongtong Cheng, Haitong Wan, Daojun Yu

**Affiliations:** ^1^ Affiliated Hangzhou First People’s Hospital, Zhejiang University School of Medicine, Hangzhou, China; ^2^ Department of Medical Laboratory, Hangzhou Women’s Hospital, Hangzhou, China; ^3^ School of Life Sciences, Zhejiang Chinese Medical University, Hangzhou, China; ^4^ Department of Medical Laboratory, Zhejiang Hospital, Hangzhou, China

**Keywords:** *Acinetobacter baumannii* (*A. baumannii*), iron ion, biofilm, human pulmonary alveolar epithelial cells (HPAEpiC), type VI secretion system (T6SS), hemolysin co-regulated protein (Hcp)

## Abstract

*Acinetobacter baumannii* is a type of bacterial nosocomial infection with severe drug resistance. Hemolysin co-regulated protein (Hcp) is a marker of activated type VI secretion system (T6SS), a key secretory system that promotes Gram-negative bacteria colonization, adhesion, and invasion of host cells. Hcp is also regulated by iron ions (Fe). In this study, an ATCC17978 *hcp* deletion strain (ATCC17978Δ*hcp*), an *hcp* complement strain (ATCC17978Δ*hcp^+^*), and an *A. baumannii*–green fluorescent protein (GFP) strain were constructed and used to investigate the role of *hcp* in bacterial adhesion to cells (human pulmonary alveolar epithelial cells (HPAEpiC)) and biofilm formation. Our results indicate that the inhibitory concentrations of the three *A. baumannii* strains (ATCC17978 wild type, ATCC17978Δ*hcp*, and ATCC17978Δ*hcp* ^+^) were drug-sensitive strains. *A. baumannii hcp* gene and iron ions might be involved in promoting the formation of a biofilm and host–bacteria interaction. Iron ions affected the ability of *A. baumannii* to adhere to cells, as there was no significant difference in the bacterial numbers when assessing the adhesion of the three strains to HPAEpiC in the presence of iron ion concentrations of 0 μM (*F* = 3.1800, *p* = 0.1144), 25 μM (*F* = 2.067, *p* = 0.2075), 100 μM (*F* = 30.52, *p* = 0.0007), and 400 μM (*F* = 17.57, *p* = 0.0031). The three strains showed significant differences in their ability to adhere to HPAEpiC. The numbers of bacteria adhesion to HPAEpiC were ATCC17978Δ*hcp*>ATCC17978Δ*hcp* ^+^>ATCC17978 in descending order. *Hcp* gene was positively regulated by iron ions in the bacteria–cells’ co-culture. It is speculated that the effect of iron ions on the interaction between *A. baumannii* and HPAEpiC might be related to the transport function of *hcp* and bacterial immune escape mechanisms.

## Introduction

*Acinetobacter baumannii* is a Gram-negative bacillus, ranking first in the pathogenic *Acinetobacter* spp (Wong et al., 2017). The incidence of multidrug-resistant and pandrug-resistant *A. baumannii* has increased over the years, and the continuous emergence of pandrug-resistant bacteria has brought serious challenges in clinical treatment (Peneş et al., 2017; Swe Swe-Han et al., 2017). Studies have shown that biofilms, protein secretion systems (Weber et al., 2017), quorum-sensing systems (Bhargava et al., 2010), and type VI secretion system (T6SS) (Chow and Mazmanian, 2010; Grandi, 2010; Hodges and Hecht, 2012; Hachani et al., 2016) exert important effects on the pathogenesis of *A. baumannii* (Repizo et al., 2015).

T6SS is a protein secretion system that was recently identified and which widely exists in Gram-negative bacilli where it can affect bacterial virulence and reduce bacterial susceptibility to antibiotics (Chow and Mazmanian, 2010; Grandi, 2010; Hodges and Hecht, 2012; Hachani et al., 2016). T6SS is also present in *A. baumannii* (Repizo et al., 2015), where it may play an important role in the process of colonization and in providing an adaptive ability advantage to mixed infections *in vivo.* A detailed analysis of T6SS-associated genes in the *Acinetobacter* genus showed an impressive genetic versatility in T6SS-associated islands, and in *A. baumannii*, the T6SS effectors were extremely diverse (Lewis et al., 2019; Repizo et al., 2019). It was also shown that the T6SS of *A. baumannii* was associated with virulence and contributes to infections in immunocompromised patients and those with implanted medical devices. Moreover, approximately one-third of the *A. baumannii* clinical isolates possessed the hemolysin co-regulated protein gene (Kim et al., 2017).

Hemolysin co-regulated protein (Hcp) is a structural component of T6SS and is also regarded as a marker of activated T6SS (Bingle et al., 2008). Some studies have shown that Hcp affects bacteria virulence, motility, and protease production (Carruthers et al., 2013; Sha et al., 2013). In addition, the expression and secretion of Hcp are affected by many factors (Weber et al., 2016), including environmental conditions such as temperature, pH value, and metal ion content (Tang et al., 2016).

Metal ion is an important component of the nutrition system of various organisms (Balasubramanian and Rosenzweig, 2008; Kehl-Fie and Skaar, 2010; Weaver and Peacock, 2011; Porcheron et al., 2013; Martins et al., 2017). It is a cofactor for various functional proteins and enzymes and plays important roles in electron transfer and redox reactions (Braun, 2001). So far, the iron metabolism system of Gram-negative bacteria is well understood. Although iron uptake varies among different *A. baumannii* strains, the iron metabolism system of *A. baumannii* is similar to that of other Gram-negative bacteria, which mainly uses heme as the source of iron (Zimbler et al., 2009). *A. baumannii* achieves iron uptake and maintains intracellular iron balance through the iron carriers, bauBABCDE, the ATP-binding cassette (ABC) transporter complex, and the ferric uptake regulator (Fur) (Yamamoto et al., 1994; Mihara et al., 2004; Runyen-Janecky, 2013; Fiester et al., 2015; Runci et al., 2019); however, whether iron ions affect the function of T6SS and biofilm formation and mediates the release of factors of bacterial virulence factors remains to be investigated.

A biofilm comprises a dense population of bacteria (Stoodley et al., 2002), and iatrogenic pathogens are prone to colonize medical devices, and therefore, the formation of a biofilm is one of the important causes of nosocomial infections (Tomaras et al., 2003). Adhesion to the host is the first stage of bacterial colonization and infection (Lee et al., 2006) and involves the formation of a biofilm, which provides a reservoir for persistent or sudden infection of the host by bacteria (Del Pozo, 2018). Studies have shown that the formation of a biofilm is regulated by the pilus assembly system (McQueary and Actis, 2011), growth temperature, and concentration of extracellular free iron (Longo et al., 2014). Therefore, we analyzed the effect of Hcp on the antibiotic susceptibility, biofilm formation, and cell adhesion capacity of *A. baumannii* by constructing an *hcp*-deficient *A. baumannii* strain (ATCC17978Δ*hcp*) and *hcp*-complementant *A. baumannii* strain (ATCC17978Δ*hcp^+^*). Meanwhile, 2,2′-dipyridyl (DIP; an iron-chelating agent) and ferric chloride (Alexandra et al., 2013; Fiester et al., 2015; Bethke et al., 2016; Aubourg et al., 2020), were added into the culture medium to generate a culture environment of different iron ions to study the effect of iron ions on the interaction of *hcp* gene between *A. baumannii* and human alveolar epithelial cells.

## Methods

### Bacterial Strains, Plasmids, and Cells

The bacterial strains and plasmids used in this study are listed in **Table S1**. *A. baumannii* ATCC17978 (ATCC17978) was used as the wild-type strain for all experiments. *Escherichia coli* and *A. baumannii* were grown in Luria–Bertani (LB) broth at 37°C. ATCC17978 and the plasmid pWH1266 were donated by Yunsong Yu from Sir Run Run Shaw Hospital, Zhejiang University School of Medicine. Human pulmonary alveolar epithelial cells (HPAEpiC) were purchased from ScienCell Research Laboratories, Inc. (CBR130583, San Diego, CA, USA) and cultured in Roswell Park Memorial Institute (RPMI) 1640 medium containing 10% fetal bovine serum (FBS) and 100 U/ml of penicillin/streptomycin (GIBCO, Grand Island, NY, USA) at 37°C and 5% CO_2_.

### Construction of the *hcp* Deletion

The primers were obtained from Tsingke Biological Technology (**Table S1**). We constructed unmarked in-frame deletions of *hcp* in ATCC17978 modified according to Oh *et al.* (Oh et al., 2015). *Hcp* gene was selected from the ATCC17978 genome for targeted deletions. The 5′ end of *hcp*-up-Mut*-*F (**Table S1**) contained a 22-bp homology arm that was complementary to the linearized pDS132. The 5′ end of *hcp*-down-Mut-R (**Table S1**) contained a 21-bp homology arm that was complementary to *tet*-F, and the 5′ end of *tet*-R (**Table S1**) contained a 20-bp homology arm that was complementary to the linearized pDS132. The upstream and downstream homologous fragments of *hcp* were ligated to the inverse PCR product of the linearized plasmid pDS132. The *tet* (tetracycline) gene fragment was amplified from the plasmid pBR322 using the Hieff Clone™ Multi One Step Cloning Kit (Yeasen, Shanghai, China) to obtain the pDS132*::hcp*-*tet* recombinant plasmid, which was used to transform into *E. coli* S17-1 *λ pir^+^* competent cells (**Table S1**). To screen and select positive transconjugants and eliminate donor cells, antibiotic resistance screening (10 μg/ml of Tet and 20 μg/ml of chloramphenicol), gel electrophoresis, and sequencing were used to verify the insertion (Hangzhou Mingke Biotechnology Co., Ltd., Hangzhou, China; Sequence Read Archive (SRA) accession: OL470900). The positive strain was named *E. coli* S17-1 *λ pir^+^* (pDS132::*hcp*-*tet*). Then, trans-conjugation was performed between *E. coli* S17-1 *λ pir^+^* (pDS132::*hcp*-*tet*) donor strain and ATCC17978 recipient strain to transfer and integrate pDS132*::hcp-tet* into the chromosome of ATCC17978.

The two strains were mixed with a ratio of 1:1 on antibiotic-free LB agar plates and cultured at 30°C for 18–24 h. A monoclonal colony was randomly selected from the colonies grown on the 100 μg/ml ampicillin (Amp) and 10 μg/ml Tet resistant plate, and the suspected positive fragments were then sequenced for verification (Hangzhou Mingke Biotechnology Co., Ltd.; SRA accession: OL470901). The positive strain was named ATCC17978Δ*hcp::tet-sacB* (this is the first homologous recombination). In addition, ATCC17978Δ*hcp::tet-sacB* was cultured on LB agar plates containing 10% sucrose and no NaCl. If ATCC17978*Δhcp*::tet-sacB cultured on LB agar plates containing 10% sucrose and no NaCl grown, it means that the second recombination event was successful. After 18–24 h, well-grown colonies were picked and simultaneously transferred to LB agar plates without antibiotics and LB agar plates with 20 μg/ml of chloramphenicol (Chl). Colonies that grew on antibiotic-free LB agar plates and did not grow on LB agar plates with 20 μg/ml of Chl were candidate target colonies. PCR and sequencing were used to verify whether the target gene was excised (Hangzhou Mingke Biotechnology Co., Ltd.; SRA accession: OL470902). A single clone with no *hcp* sequence was saved as ATCC17978Δ*hcp*.

### Construction of *hcp* Reconstituted Strains

Complementation was performed using the plasmid pWH1266 (Swe Swe-Han et al., 2017). The amplification of the *hcp* gene sequence that was obtained by *Sal*I and *Bam*HI enzyme restriction was cloned into a linearized plasmid pMD19-T that was digested by *Sal*I and *Bam*HI enzymes to obtain the recombinant plasmid pMD19-T*::hcp*. Then, pWH1266*::hcp* was constructed in the same way while making the template pMD19-T*::hcp* to obtain the *hcp* gene sequence. pMD19-T*::hcp* and pWH1266*::hcp* reconstitute plasmids were both introduced into *E. coli* DH5α to generate the strain *E. coli* DH5α (pMD19-T*::hcp*) and *E. coli* DH5α (pWH1266*::hcp*). Then, trans-conjugation was performed between *E. coli* DH5α (pWH1266*::hcp*) donor strain, ATCC17978Δ*hcp* recipient strain, and *E. coli* HB101 (pRK2013) helper strain to transfer and integrate pWH1266*::hcp* into ATCC17978Δ*hcp*. The three strains were mixed at a ratio of 2:1:1 (donor bacteria:recipient bacteria:helper bacteria), spread on antibiotic-free LB agar plates, and cultured overnight at 28°C. An appropriate number of bacteria were scraped and mixed with 1 ml of LB medium without antibiotics, and an appropriate amount was then spread onto plates with 100 μg/ml of Amp and 100 μg/ml of ticarcillin (Tic). The *hcp*-reconstituted strain named ATCC17978Δ*hcp^+^* was selected on LB agar containing 100 μg/ml of Amp and 100 μg/ml of Tic and further verified by PCR and sequencing (Hangzhou Mingke Biotechnology Co., Ltd.; SRA accession: OL470903).

### Construction of the *Acinetobacter baumannii*–Green Fluorescent Protein Strain

To better explore the function of *A. baumannii* ATCC17978 *hcp* gene, the plasmid pET-RA was introduced into ATCC17978 wild type, *hcp* deletion (ATCC17978Δ*hcp*), and *hcp*-reconstruction (ATCC17978Δ*hcp* ^+^) strains. The plasmid pET-RA was used in this study with some modifications based on a previous publication in the National Center for Biotechnology Information (NCBI) (GenBank: HM219006). The plasmid pET-RA was introduced into ATCC17978 wild type, ATCC17978Δ*hcp*, and ATCC17978Δ*hcp* ^+^ strains using the triparental conjugative transfer method according to Carruthers et al. (2013). First, the pET-RA plasmid was introduced into *E. coli* DH5α competent cells and cultured overnight on an LB agar plate with 100 μg/ml of rifampicin (Rif) for resistance screening. *E. coli* DH5α (pET-RA) was verified by PCR and sequencing (Hangzhou Mingke Biotechnology Co., Ltd.; SRA accession: OK655938). Then *E. coli* DH5α (pET-RA) was used as a donor strain, the three *A. baumannii* strains (ATCC17978 wild type, ATCC17978Δ*hcp*, and ATCC17978Δ*hcp* ^+^) were used as recipient strains, and *E. coli* HB101 (pRK2013) was used as a helper strain to transfer the pET-RA into the three *A. baumannii* strains. The pET-RA-reconstituted strains, ATCC17978–green fluorescent protein (GFP), ATCC17978Δ*hcp*-GFP, and ATCC17978Δ*hcp^+^*-GFP, were selected on LB agar containing 100 μg/ml of Amp and 10 μg/ml of Rif and verified by PCR and sequencing (Hangzhou Mingke Biotechnology Co., Ltd.; SRA accession: OK655938).

### Antimicrobial Susceptibility Test

A fully automatic bacterial identification instrument (VITEK2-COMPACT, bio-Mérieux, Marcy-l’Étoile, France) was used to test the antibiotic susceptibility of ATCC17978, ATCC17978Δ*hcp*, and ATCC17978Δ*hcp^+^*, according to the instructions of the VITEK 2 AST Gram-negative card.

### Growth Curves

The three strains (ATCC17978, ATCC17978Δ*hcp*, and ATCC17978Δ*hcp^+^*) were inoculated onto plates and cultured for 18–24 h. Single colonies were picked for amplification, inoculated into 100 ml of LB liquid culture medium at a 1:100 ratio, and cultured on a shaker at 37°C. The blank control was LB medium without the addition of bacteria. Bacterial culture medium samples of optical density (OD) at 600 nm (OD_600_) were measured on a microplate reader every 0.5 h for the first 6 h and then measured every 1 h in a period of 6–16 h.

### Biofilm Assay

Biofilms were measured by a crystal violet staining assay (Stepanović et al., 2010). Briefly, a single colony of the three strains, ATCC17978, ATCC17978Δ*hcp*, and ATCC17978Δ*hcp^+^*, was picked and cultured in 5 ml of LB medium for 18 h. The bacterial culture was normalized to 0.5 McFarland standard and diluted with fresh LB medium (1:20) containing different iron ion concentrations: 0 (iron deprivation: the media and reagents were treated by adding 100 μM of the iron ion chelator, DIP), 25, 100, 200, 400, and 800 μmol/L. The iron content of the media and reagents used in all assays was taken into account. According to the initial concentration of iron in the bacteria/cell culture medium, the final concentration of iron in the medium was adjusted to 25, 100, 200, 400, and 800 μmol/L with ferric chloride solution. Flat-bottomed microtiter 96-well plates, 12-well culture plates, and 5-ml polystyrene tubes were filled with 200 μl, 2 ml, and 2 ml of the bacterial dilution, respectively. Tubes containing only LB were included in the test as negative controls. The bacterial culture in the polystyrene tubes was cultured for 24 h at 37°C and 180 rpm/min (on a shaker), while the bacterial culture in the 96-well plates and 12-well plates was cultured without shaking. The OD_600_ was read on a 96-well bacteria microplate reader. Planktonic cells were removed, and then each tube and well was washed 3 times with phosphate-buffered saline (PBS; pH 7.4) to remove all non-adherent bacteria. After fixation with 4% paraformaldehyde for 10 min and air-drying, the remaining attached bacteria were stained with 0.1% crystal violet (w/v) at room temperature for 20 min. Subsequently, the dye was removed, and the wells and tubes were washed 3 times with PBS followed by air-drying. For quantification, the dye was released from the bacteria by incubation in 250 μl of 95% ethanol for 20 min, and the optical density at 580 nm (OD_580_) was measured on a microplate reader (Tucker et al., 2014). The OD_580_/OD_600_ was used in the statistical analysis for the wells’ biofilm. The formation of a purple ring at the junction bacterial culture–air in the polystyrene tube and pellicles floating on the surface of the culture medium in the 24-well culture plates were used for semiquantitative analysis (the symbols −, −/+, ++, +++, and ++++ were used to indicate the amount of ring purple formation).

### Interaction of Bacteria With Human Pulmonary Alveolar Epithelial Cells Utilizing Real-Time Cell Analysis

To determine whether *hcp* gene and iron ions would influence the interaction between bacteria and HPAEpiC (Cat. No. CBR130583, ScienCell Research Laboratories, Inc., San Diego, CA, USA), the interaction between bacteria and HPAEpiC was analyzed by real-time cell analysis (RTCA) (Chen et al., 2019). The RTCA system was consisted of an RTCA single plate station, an RTCA computer with integrated software (version 1.1; Roche Applied Science, Mannheim, Germany), and disposable 96-well E-plates of the xCELLigence system (Cardio Plate 96; ACEA Biosciences, San Diego, CA, USA). RTCA is a system that is based on electronic detection of biological processes that allow label-free, real-time, and continuous monitoring of cellular adhesion, proliferation, growth, and morphology (Stefanowicz-Hajduk and Ochocka, 2020). RTCA has initially been developed to detect variations in the impedance signal [expressed as cell index (CI)] due to the attachment and growth of adherent eukaryotic cells on the gold microelectrodes placed in the bottom of the E-plate, which has a surface equivalent to 96-well standard microplates. The CI was recorded every 10 min during the incubation period (Diana et al., 2016; Diana et al., 2017). A special scaled index, defined as the normalized cell index (NCI), was used to minimize the influence of inter-experimental variations. The NCI was calculated as the ratio of the CI value at a given time-point to the initial CI value at the time of exposure and used in the analysis to represent baseline cell activity (Peng et al., 2017). HPAEpiC was cultured to 80%–90% confluence and plated onto 16-well plates for RTCA at 2 × 10^4^ cells/well. RPMI containing 0 (iron deprivation, the RPMI was treated by adding DIP), 25, 100, 400, or 800 μmol/L iron ion was used to detect whether iron ions affect cell growth and to choose the concentration of iron ions for follow-up experiments (the used media and reagents’ iron content this assay was taken into account). After 24-h incubation of HPAEpiC at 37°C in 5% CO_2_ for 24 h, two experimental settings were performed. On the one hand, ATCC17978, ATCC17978Δ*hcp*, or ATCC17978Δ*hcp^+^* was added to the rinsed plates at a multiplicity of infection (MOI) of 100 in fresh RPMI 1640 medium; and on the other hand, RPMI containing 0 (iron deprivation), 25, 100, and 400 μmol/L of iron ions with ATCC17978 or ATCC17978Δ*hcp* was added to the washed cell plates. Then cell culture was continued for 24 h. Before and after the addition of the bacteria, changes in CI were continuously observed by RTCA.

### Adhesion and Invasion Assays of Human Pulmonary Alveolar Epithelial Cells

The experimental procedures were developed according to previous studies (Choi et al., 2008; Wu et al., 2014), and the optimal time for bacteria–cell co-culture was determined in conjunction with the RTCA results to investigate the effect of *hcp* on HPAEpiC bacterial adhesion and invasion.

HPAEpiC was cultured to 80%–90% confluence and added to 12-well cell culture plates, with or without coverslips (coverslips were treated with 0.1 mg/ml of polylysine solution), at a concentration of 2 × 10^4^ cells/well. After 24-h incubation at 37°C in 5% CO_2_, ATCC17978, ATCC17978Δ*hcp*, or ATCC17978Δ*hcp^+^* was added to the rinsed cell plates at a MOI of 100, and with different concentrations of iron ions (0, 25, 100, and 400 μmol/L). According to the RTCA results, the growth index of the bacteria–cell co-culture had the most significant changes at approximately 5 h of co-culture. Therefore, 5 h was chosen as the duration of bacteria–cell co-culture, and GFP-labelled strains were used in groups designed for experiments using fluorescence microscopy.

After 5 h of bacteria–cell co-culture, the coverslip group was washed three times with PBS, fixed in 4% paraformaldehyde at room temperature for 20 min, and then washed three times with PBS. For the GFP strain, it was permeabilized with 0.1% Triton X-100 for 5 min, washed three times with PBS, and treated with 1× iFluor™ 647-labeled phalloidin, which was protected from light for 1 h at room temperature and then washed three times with PBS. The cell nucleus was re-stained with 1 μg/ml of 4′,6-diamidino-2-phenylindole (DAPI) solution, stained for 10 min in the dark at room temperature, and washed three times with PBS and one time with sterile distilled water. Finally, the slides were stained with Fluoroshield™. Gram staining strains (ATCC17978, ATCC17978Δ*hcp*, and ATCC17978Δ*hcp^+^*) were observed under ordinary light microscopy, and the GFP strains were observed and imaged using the Olympus fluorescence microscope (IX71). Four replicate wells were set for each experiment. After 5 h of co-culture, the cells were rinsed with PBS to remove planktonic bacteria. One well was treated with 0.25% trypsin, and the suspension was collected in a centrifuge tube, followed by the addition of 100 μl of FBS to neutralize trypsin, and then the number of cells was counted after resuspension. The remaining three wells of cells were treated with PBS containing 1% Triton X-100 for 10 min to release intracellular bacteria. The suspension was collected in a centrifuge tube, followed by gradient dilution. The diluted solutions were inoculated onto LB agar plates (dilute to 10^−3^), and the same dilution was repeated 3 times. Bacteria were incubated overnight at 37°C in 5% CO_2_, and the number of bacterial colonies was calculated the next day. The number of cells was used as a basis for comparing the number of bacteria and normalized to obtain a standardized number of bacteria for statistical analysis.

### Expression of *hcp*

To explore changes in *hcp* expression when bacteria were cultured in different environments, including with or without cells, and with different iron ions (0, 25, 100, 200, 400, and 800 μmol/L), ATCC17978 and ATCC17978Δ*hcp* bacterial suspensions were added to the fresh medium of the different culture conditions.

HPAEpiC was cultured to 80%–90% confluence, plated into 12-well cell culture plates at a concentration of 2 × 10^4^ cells/well, and cultured for 24 h at 37°C in 5% CO_2_. ATCC17978 or ATCC17978Δ*hcp* bacterial suspensions were added at a MOI of 100 to a fresh medium containing different iron ion concentrations (0, 25, 100, and 400 μmol/L), with or without HPAEpiC. For bacteria cultured with HPAEpiC, after 24-h co-culture at 37°C, the culture supernatant was discarded, and the HPAEpiC was rinsed six times with PBS to remove planktonic bacteria. The RNA from each group of bacteria was extracted and subjected to reverse transcription–quantitative PCR (RT-qPCR). For bacteria only, bacteria were cultured 24 h at 37°C and with 180 rpm/min shaking. RNA was then extracted from each group of bacteria using the Bacteria RNA Extraction Kit (Nanjing Vazyme Medical Technology Co., Ltd., Nanjing, China), and the AceQ^®^ qPCR Probe Master Mix (Nanjing Vazyme Medical Technology Co., Ltd.) was used for RT-PCR (ABI7500) to detect the relative expression of *hcp* gene in *A. baumannii* under different culture conditions. The used primer and probe information is provided in the **Supplementary Material** (**Table S2**). *gyrB* gene was used as a reference gene (Teixeira et al., 2017), and a nucleic acid extract of ATCC17978Δ*hcp* was used as a negative control.

### Quantification and Statistical Analysis

The data were expressed as the mean ± SD. One-way ANOVA was used to statistically analyze the effect of different iron ion concentrations on bacterial biofilm formation (OD_580/_OD_600_), the number of adherent bacteria (normalized to colony-forming unit (CFU)), and the relative expression of bacterial *hcp* during bacterial culture and bacteria–cell co-culture. Two-way ANOVA was used to statistically analyze the quantitative results of biofilm formation, the number of adherent bacteria of different strains under the influence of iron, and the effect of HPAEpiC excitation on bacterial *hcp* expression. When analyzing the effect of different iron ion concentrations on *hcp* expression, 2^−ΔΔCt^ was used for the statistical analysis (Santangelo et al., 2012; Chen et al., 2019). *p* < 0.05 indicated statistical significance, while 2^−ΔCt^ was used for analyzing the effect of HPAEpiC excitation on bacterial *hcp* expression (Santangelo et al., 2012; Chen et al., 2019).

## Results

### Antibiotic Susceptibility Test

The result of the antibiotic susceptibility test showed that the inhibitory concentration of trimethoprim/sulfonamide for ATCC17978Δ*hcp* is only one concentration gradient different and that the susceptibility of ATCC17978Δ*hcp^+^* to trimethoprim/sulfonamide is similar to that of the wild-type strain. The inhibitory concentrations of other antibiotics on the two other strains (ATCC17978Δ*hcp and* ATCC17978Δ*hcp^+^*) did not increase, suggesting that the three strains were *A. baumannii* drug-sensitive strains (**Table 1**).

**Table 1:** Antibiotic susceptibility test (MIC, mg/L).

Strains	TGC	SXT	AMP	AMC	CZ	CX	FEP	CRO	IPM	GM	TM	CIP	LVX	FD
ATCC17978	≤0.5	160	16	4	64	64	2	8	1	1	1	0.5	≤0.25	≥512
ATCC17978*Δhcp*	1	320	16	4	64	64	4	16	1	1	1	1	1	≥512
ATCC17978*Δhcp^+^*	≤0.5	160	32	8	64	64	4	16	1	1	1	1	1	≥512

TGC, tigecycline; SXT, trimethoprim/sulfonamide (2:1); AMP, ampicillin; AMC, amoxicillin/clavulanic acid (2:1); CZ, cefazolin; CX, cefoxitin; FEP, cefepime; CRO, ceftriaxone; IPM, imipenem; GM, gentamicin; TM, tobramycin; CIP, ciprofloxacin; LVX, levofloxacin; FD, nitrofurantoin.

### The Role of *hcp* Gene and Iron Ions in Biofilm Formation of *Acinetobacter baumannii*

**Figure 1:**
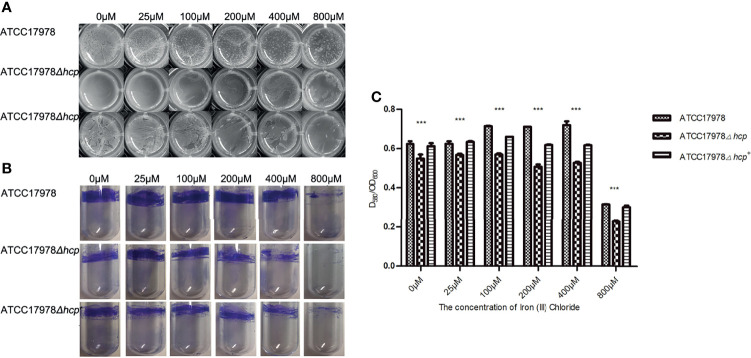
Roles of *hcp* gene and iron ions in ATCC17978, ATCC17978Δ*hcp*, and ATCC17978Δ*hcp^+^* biofilm formation. **(A)** Biofilm formation in 24-well cell culture plates. **(B)** Crystal violet staining of biofilm formation rings in 5-ml polystyrene tubes. **(C)** Ratio of optical density (OD) of biofilm formation measured by crystal violet staining and growth turbidity (OD_580_/OD_600_) (the asterisks indicate a significant difference in biofilm formation ability between ATCC17978 and ATCC17978Δ*hcp* strains at the same iron ion concentration). ^***^ *p* < 0.0001.

The ability to form biofilms on an abiotic surface in the different culture conditions of ATCC17978, ATCC17978Δ*hcp*, and ATCC17978Δ*hcp^+^* is shown in **Figure 1** and **Table 2**. The construction processes of ATCC17978Δ*hcp* and ATCC17978Δ*hcp^+^* are shown in **Figure S1**. Pellicles floating on the surface of the culture medium in 24-well culture plates (**Figure 1A**) and crystal violet staining of the biofilm formation rings in polystyrene tubes (**Figure 1B**) showed that ATCC17978Δ*hcp* exhibits a significantly decreased ability to form a biofilm as compared to that of ATCC17978, while ATCC17978Δ*hcp^+^* regained this ability.

**Table 2:** The ability of biofilm formation among different *Acinetobacter baumannii* strains and different concentrations of iron ion.

The concentration of iron ion (μmol/L)	ATCC17978	ATCC17978Δ*hcp*	ATCC17978Δ*hcp^+^*
0	++++/*++++	++/*++	++++/*+++
25	++++/*++++	++/*+++	++++/*+++
100	+++++/*++++	++/*+++	+++++/*+++
200	+++/*++++	+/*++	+++/*++
400	+++/*++	+/*+	+++/*++
800	+++/*+	±/*±	++/*+

For example, ±/*± indicates few formations. ++++/*++++ indicates that pellicles floating on the surface of the culture medium increased, surface layering became apparent, and the structure is more complicated than +++, or purple ring at the junction of the bacterial culture in polystyrene tubes was obviously increased compared to *+++. +, indicates the ability of biofilm formation. * indicates formation of a purple ring at the junction of the bacterial culture in polystyrene tubes.

With the increase of iron ion concentration (0–100 μM with 0 μM corresponding to iron deprivation), the formation of pellicles increased, the surface layering became apparent, and the structure became complicated. With a further increase in iron ion concentration (200–800 μM), the formed biofilm gradually became lumpy or fragmented. Regardless of the changes in iron ion concentration, the biofilm formation of the ATCC17978Δ*hcp* strain was less than that of the ATCC17978 strain (**Figure 1A**). In addition, crystal violet staining showed that the biofilm formation rings in the polystyrene tube increased at first and then decreased with a further increase in iron ion concentration (0–800 μM). When the iron ion concentration reached 800 μM, the number of biofilm formation rings became the least, and among the range of 0–800 μM of iron ion, each strain exhibited a similar trend of changes in biofilm formation with the change in iron ion concentration (**Figure 1B**).

Statistical analysis using the two-way ANOVA showed that the differences (ratio of optical density of biofilm formation measured by crystal violet staining and growth turbidity, OD_580_/OD_600_) among the three strains were significant (*F* = 2336, *p* < 0.0001) (**Figure 1C**). The amount of ATCC17978 and ATCC17978Δ*hcp^+^* biofilms formed was significantly greater than that of the ATCC17978Δ*hcp*. One-way ANOVA showed that the concentration of iron ions is an important factor that could affect the biofilm formation ability of the three strains (**Figure 1C**). An increase in biofilm formation of each strain was observed at 0–100 μM of iron ion concentrations, which was followed by a decrease at 200–800 μM and a significant reduction at a concentration of 800 μM. These results were consistent with the results of the test tube method (**Figure 1B**). The *hcp* mutant did not exhibit a growth defect (**Figure S2**), indicating that the effect on the biofilm formation among ATCC17978, ATCC17978Δ*hcp*, and ATCC17978Δ*hcp^+^* did not result from a change in growth.

### *Acinetobacter baumannii* Can Infect Human Pulmonary Alveolar Epithelial Cells

**Figure 2:**
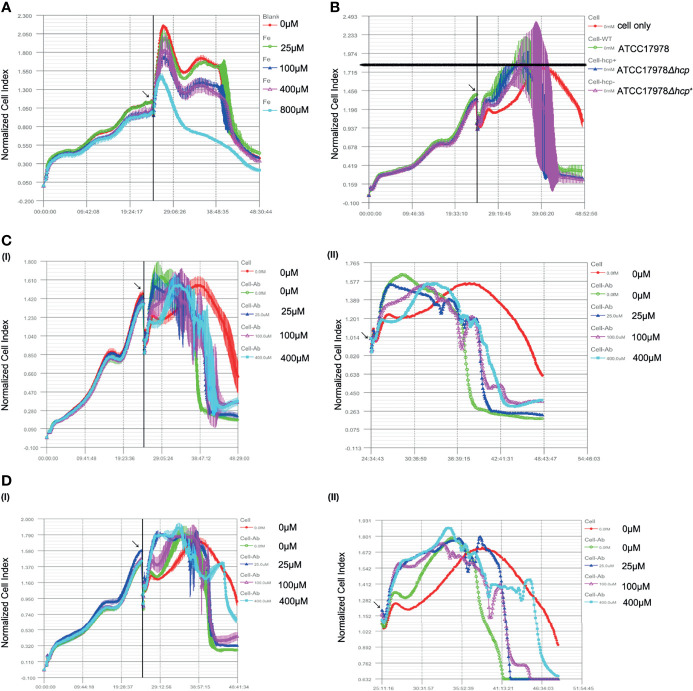
Effect of *hcp* or iron ions on the interaction between *Acinetobacter baumannii* and human alveolar epithelial cells. **(A)** Effect of different concentrations of iron ions on human pulmonary alveolar epithelial cells (HPAEpiC) growth. Cells grew well, and little difference is observed among the groups when the iron ions were less than 400 μM, while the peak normalized cell index (NCI) of the 800 μM group was the lowest. Thus, 0 (iron deprivation), 25, 100, and 400 μM were suitable for subsequent bacteria cells’ co-culture studies. **(B)** Effect of ATCC17978, ATCC17978Δ*hcp*, and ATCC17978Δ*hcp^+^* on HPAEpiC growth. As bacteria were added into culture, an increase of NCI was induced as compared to the control group (cell only). Meanwhile, the peak NCI of experimental groups appeared earlier than that of the control group. However, the peak value among the groups was similar. [**(C) (I, II)**] Effect of iron ions on the cell growth index in the co-culture of the ATCC17978 strain and the cells. Initial bacteria-induced increase in NCI and an advanced peak as compared to the control group. The 0 μM (iron deprivation), 25 μM, 100 μM, and 400 μM iron ion groups had similar curves. The lower iron ion concentration was the earliest peak. [**(D) (I, II)**] Effect of iron ions on the cell growth index in the co-culture of ATCC17978Δ*hcp* and the cells. Similar to panel [**(C (I, II)**], the initial bacterial infection induced an increase in NCI and an early peak. The curve of the 0 μM iron ion (iron deprivation) group declined faster, and there was no significant difference among experimental groups in the peak value. Contrary to panel [**(C) (I, I)**], the 0 μM, 25 μM, 100 μM, and 400 μM iron ion groups had similar curves.

The effect of *hcp* or iron ions on HPAEpiC in a bacterial–cell co-culture was observed by RTCA. As shown in **Figure 2A**, the cells grew well when the iron ion concentration was less than 400 μM, while the CI of the 800 μM iron ion group was the lowest and decreased faster than that of other groups. Moreover, there was no platform period, suggesting that 0 (iron deprivation), 25, 100, and 400 μM are suitable for subsequent bacteria–cell co-culture studies.

As shown in **Figure 2B**, with the addition of bacteria to HPAEpiC and co-culture for approximately 5 h, the cell growth curve of each group (ATCC17978, ATCC17978Δ*hcp*, and ATCC17978Δ*hcp^+^*) showed a stable exponential increase. This increase was concomitant with a significant increase in the cell growth curve of the bacteria group compared to that of the control group (cells only). However, the NCI of the bacteria group began to rapidly decline until reaching baseline, while HPAEpiC in the control group grew normally until depletion of nutrients in the culture. Otherwise, the peak value of the NCI in the pure cell culture group was delayed compared with that in the experimental groups, but the peak value of NCI in each group was approximatively 1.8.

The co-culture of ATCC17978 and HPAEpiC showed that the NCI has initially similar increasing trends between the 0 μM (iron deprivation) and 25 μM iron ion groups; however, a subsequent early decline was observed in the 0 μM iron ion group compared to that of the other experimental groups. The cell growth curves of the 100 μM and 400 μM iron ion groups had similar peak time and peak height [**Figures 2C(I, II)**]. The results of the co-culture of ATCC17978Δ*hcp* with HPAEpiC showed that the cell growth index increases and reaches the peak in advance with the addition of 25, 100, and 400 μM of iron ion concentrations. In the 0 μM iron ion group, the cell growth index slowly increased, and the cell growth curve decreased earlier [**Figure 2D(I, II)**]. The growth curve index of the co-culture of ATCC17978Δ*hcp^+^* and HPAEpiC was similar to that of ATCC17978 and HPAEpiC.

### Effect of Iron on *Acinetobacter baumannii* Adhesion and Invasion of Human Pulmonary Alveolar Epithelial Cells

**Figure 3:**
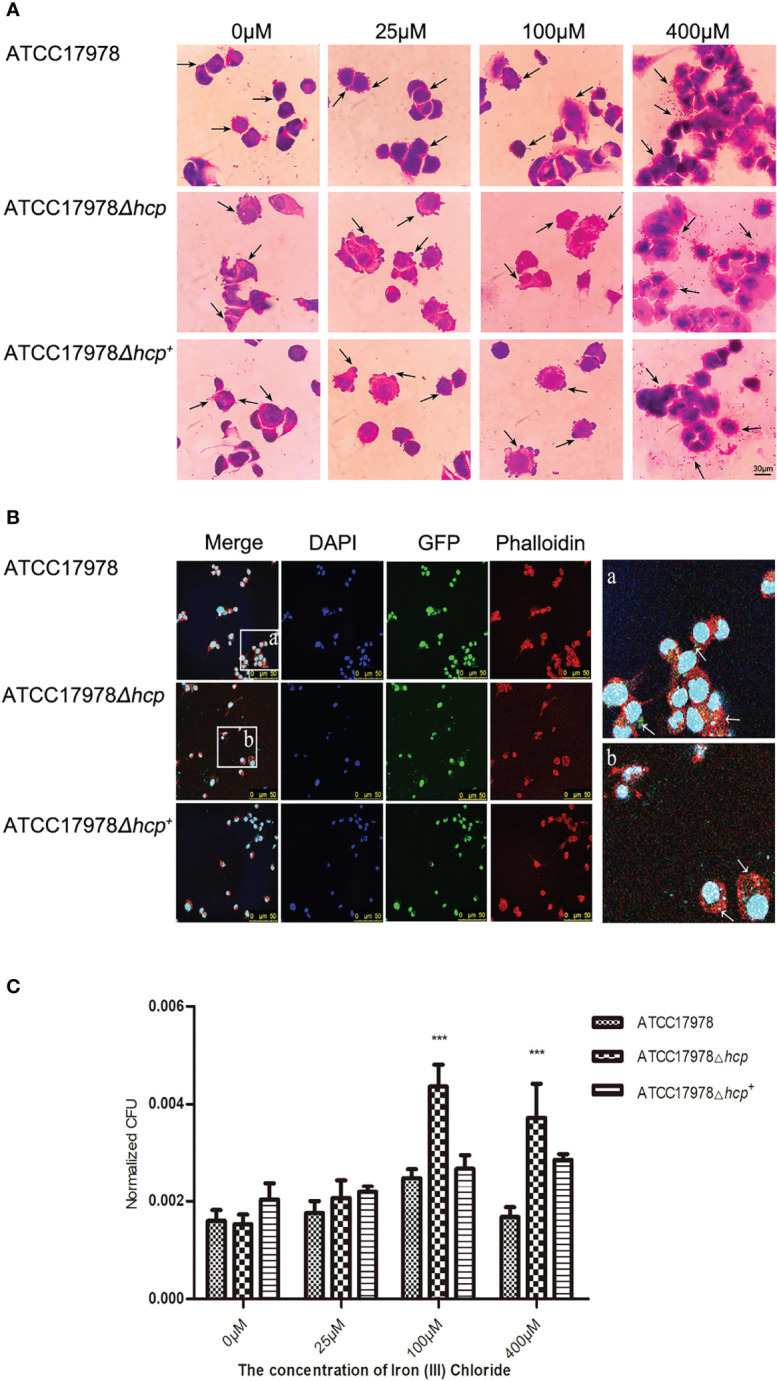
Effect of iron on the *Acinetobacter baumannii* adhesion and invasion of human pulmonary alveolar epithelial cells (HPAEpiC). **(A)** Bacterial adhesion and invasion of the cells by the three strains (ATCC17978, ATCC17978Δ*hcp*, and ATCC17978Δ*hcp^+^*) in the presence of different concentrations of iron ions was observed under a conventional microscope. The arrows point to bacteria attached to the cell surface (not all marked, ×1,000). Scale bar = 30 μM. **(B)** Fluorescence microscopy of the effect of *hcp* on bacterial adhesion and invasion of the cells. The blue fluorescence represents cellular nucleic acids that were stained with DAPI; green fluorescent protein (GFP) was the fluorescent marker of bacteria, and the red fluorescence corresponds to a phalloidin staining of cellular actin, ×400. Schematic diagram of bacterial adhesion and invasion of HPAEpiC. The GFP labeling is indicated by arrows (not all marked). **(C)** The results of the bacteria count for bacteria adhesion and invasion of HPAEpiC by the three strains at different concentrations of ferric ion. *** represents means that are under the same iron ion. Compared with ATCC17978, ATCC17978*Δhcp* has a significant difference in normalized CFU adhesion and invasion of HPAEpiC (*p* < 0.001).

We examined whether the concentration of iron ions affects *hcp* expression and investigated whether it has an influence on the bacterial adhesion and invasion of HPAEpiC by staining and bacteria growth experiments. As shown in **Figure 3A**, the morphology of the bacteria that adhered to the periphery of the cells was complete, while the morphology of the bacteria that were captured by the cells was irregular, mostly appearing as dark purple chunks or granules. Microscopic observation revealed that with the increase of iron ion concentration, the number of bacteria that adhered to the surface or entered the cells gradually increased in the 0–400 μM groups. With an iron ion concentration of 400 μM, the number of cells was greater than that in other groups. There was an increase in scattered bacteria between cells, bacteria aggregation, and adhesions between bacteria, and between bacteria and cells (**Figure 3A**).

The adhesion ability of *A. baumannii*-GFP strains to HPAEpiC was observed under an inverted fluorescence microscope. As shown in **Figure 3B**, we could not discern the difference in the bacterial number of the three strains (wild-type strain, deletion strain, and complementant strain) and their adhesion and invasion of HPAEpiC by inverted fluorescence microscopy.

As shown in **Figure 3C**, there were significant differences in the ability of the strains to adhere HPAEpiC (different strains: *F* = 30.46, *p* < 0.0001; different iron ion concentrations: *F* = 37.85, *p* < 0.0001). The 0 μM iron ion (iron deprivation) group (*F* = 3.1800, *p* = 0.1144) and the 25 μM iron ion group (*F* = 2.067, *p* = 0.2075) had *p*-values that were greater than 0.05, indicating that there was no significant difference in the bacterial numbers of the three strains during their adhesion and invasion of HPAEpiC. For the 100 μM iron ion (*F* = 30.52, *p* = 0.0007) and 400 μM iron ion (*F* = 17.57, *p* = 0.0031) groups, the three strains showed significant differences in their ability to adhere and invade HPAEpiC (**Figure 3C**). The number of adhering bacteria to HPAEpiC was in the following descending order: ATCC17978Δ*hcp*>ATCC17978Δ*hcp^+^* >ATCC17978.

### Effect of Culture Conditions on *Acinetobacter baumannii hcp* Expression

**Figure 4:**
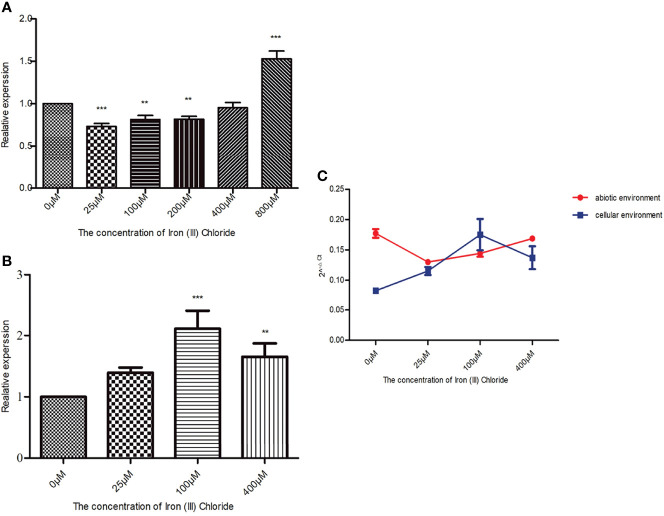
Effect of culture conditions on *Acinetobacter baumannii hcp* expression. The final concentrations of iron ion supplementation in the abiotic environment were as follows: 0 (iron deprivation), 25, 100, 200, 400, and 800 μM, while 0 (iron deprivation), 25, 100, and 400 μM iron ion concentrations were used in the cellular environment. The relative changes of **(*A*)** *baumannii hcp* expression in the presence of different iron ion concentrations while bacteria were cultured in the cellular or abiotic environment were calculated by the 2^−ΔΔCt^ method. *gyrB* gene was used as an internal reference, and *hcp* expression in the 0 μM group was 1. The 2^−ΔCt^ method was used to compare the expression of *hcp* between the cellular and abiotic environments. **(A)** Effect of different iron ion concentrations on *A baumannii hcp* expression when cultured in the abiotic environment. **(B)** Effect of different iron ion concentrations on *A baumannii hcp* expression during *A baumannii*-cells’ co-culture. **(C)** Changes in *hcp* expression in the cellular and abiotic environments. The asterisks indicate significant differences in *hcp* expression for bacteria in other iron ion concentration groups relative to the 0 μM iron ion (iron deprivation) group, **p < 0.01, ***p < 0.001.

When in an abiotic environment, *hcp* relative expression in the 800 μM iron ion group was 1.5 times higher than that in the control group, which was the highest among the experimental groups. Dunnett’s multiple comparison analysis showed that compared with the 0 μM iron ion (iron deprivation) group, the *q*-values of the remaining five groups were 6.283 (25 μM), 4.396 (100 μM), 4.317 (200 μM), 1.092 (400 μM), and 12.53 (800 μM). Except for the 400 μM iron ion group, the *p*-values of the other groups were less than 0.05, and therefore, the differences were significant (**Figure 4A**).

In a cellular environment, the relative expression of *hcp* in the experimental groups was increased compared with the control group (iron deprivation group), and *hcp* expression of *A. baumannii* among all the experimental groups was statistically significant (*F* = 18.73, *p* = 0.0006). According to Dunnett’s multiple comparison analysis, the *q* values of the other three groups were 2.573 (25 μM), 7.290 (100 μM), and 4.293 (400 μM) when compared with the 0 μM iron ion group. The difference in the *hcp* expression between the 100 μM and 0 μM iron ion groups was approximately 2 (**Figure 4B**).

**Figure 4C** shows that the relative expression of *hcp* is significantly different from that of *A. baumannii* co-culture with HPAEpiC (*F* = 34.33, *p* < 0.0001) and that, except for the 25 μM iron ion group, the differences are significant in *hcp* relative expression in the cellular and abiotic environments (**Table 3**). In addition, except for the 100 μM iron ion group, the relative expression of *hcp* in the abiotic environment was greater than that in the cellular environment.

**Table 3:** Expression of *hcp* of *Acinetobacter baumannii* in different culture environments (2^−ΔCt^, means ± SD).

Groups (μM)	Cellular environment	Abiotic environment	*t*	*p*
0	0.0829 ± 0.001	0.177 ± 0.007	9.568	*p* < 0.001
25	0.1159 ± 0.007	0.130 ± 0.001	1.498	*p* > 0.05
100	0.1759 ± 0.026	0.144 ± 0.005	3.134	*p* < 0.05
400	0.1379 ± 0.019	0.169 ± 0.004	3.208	*p* < 0.05

The iron ion supplementation final concentrations in abiotic environment were as follows: 0, 25, 100, and 400 μM. 2^−Ct^ method was used to compare the expression of hcp between cellular environment and abiotic environment. Except for the 25 μM iron ion group, the differences in hcp relative expression in cellular and abiotic environments were significant. Additionally, with the exception of the 100 μM iron ion group, the relative expression of hcp in the abiotic environment was greater than that in cellular environment.

## Discussion

In this study, an *A. baumannii* ATCC17978 *hcp* deletion strain was constructed to investigate the role of *hcp* in bacterial adhesion to cells and biofilm formation. An ATCC17978 *hcp* complement strain was also constructed to verify *hcp* gene function. To explore the changes in *A. baumannii hcp* expression when co-cultured with HPAEpiC in abiotic and cellular environments, TaqMan RT-qPCR was used to detect the relative expression of *hcp* gene in the wild-type strain. The results showed that *hcp* expression is downregulated when the bacteria and cells (HPAEpiC) are co-cultured in a cellular environment and when compared with the co-culture in an abiotic environment. These results suggest that when *A. baumannii* is co-cultured with HPAEpiC, *hcp* expression is adaptively downregulated to avoid the stress resistance of alveolar epithelial cells to *A. baumannii* through reducing Hcp synthesis, T6SS activity, and virulence factor secretion. These effects weaken the resistance of alveolar epithelial cells (hosts) to *A. baumannii* and improve bacteria’s survival rate (German et al., 2016; Lobato-Marquez et al., 2016; Kim et al., 2017).

Studies have shown that antibiotic-resistant plasmids encoding tetracycline resistance (TetR)-related regulatory proteins exist in *A. baumannii*. The presence of antibiotic-resistant plasmids might silence T6SS function and increase bacterial resistance. Moreover, the elimination of antibiotic-resistant plasmids leads to T6SS activation (Weber et al., 2015), suggesting that there is a negative regulation between T6SS activation and bacterial resistance to some antibiotics. Kim et al. found that *tetR* can be detected in strains that do not express Hcp and that *hcp*-negative strains have an increased resistance to antibiotics, including tigecycline (Kim et al., 2017). In this study, the sensitivities of the three strains, ATCC17978Δ*hcp*, ATCC17978, and ATCC17978Δ*hcp^+^*, to Gram-negative bacilli conventional antibiotics were measured. The results showed that compared with those of ATCC17978, the inhibitory concentrations of other antibiotics on the two other strains (ATCC17978Δ*hcp and* ATCC17978Δ*hcp^+^*) did not increase, as there was only one concentration gradient difference. These results suggest that the three *A. baumannii* strains were drug sensitive and indicate that the ATCC17978 strain has a T6SS activity and expresses Hcp (Weber et al., 2015).

The formation of a biofilm is regulated by different factors. In this study, the three strains’ biofilm formation was generated under different concentrations of iron ions. Biofilm formation of the strains is regulated by iron ions, with the highest biofilm formation observed at a concentration of 100 μM and its abolition at an iron ion concentration of 800 μM.

Studies have shown that iron metabolism is closely related to the formation of a biofilm. Iron deficiency inhibits the formation of a biofilm, while its increase promotes the formation of a biofilm (Singh et al., 2002; Kumar et al., 2017). In an iron deficiency environment, the formation of a biofilm depends on the regulation of bacterial iron ion acquisition systems.

Sufficient iron ions promote the formation of a biofilm, while the expression of *hcp* is inhibited. When the concentration of iron ions is too high, it can inhibit the formation of a biofilm, which may be related to the toxicity of a high concentration of iron ions in cells. In addition, the presence of *hcp* is beneficial for the formation of a biofilm. It can be inferred that under sufficient availability of free iron ions in the external environment, bacteria biofilm formation occurs at a high level, and therefore, there was no need to mobilize additional iron acquisition channels.

In addition, we found that the formation of a biofilm mainly depends on iron ions in the biological environment. From the results of the Gram staining of the bacteria–cells’ co-culture, we found that at 400 μM of iron ions, there was a presence of bacterial colonies and other substances related to intercellular biofilm formation, which had the appearance of a pink network after Gram staining, and the initiation of biofilm formation. In contrast, no signs of biofilm formation appeared at a low iron ion concentration. This is consistent with the report of Singh and his colleagues (Singh et al., 2002) that showed that host cells have a natural immune response to bacterial biofilm formation. By competitively binding iron in the environment, host cells can limit the use of iron by bacteria, preventing the formation of small colonies and, thus, inhibiting the formation of a biofilm.

In this study, the effect of iron ions on bacterial adhesion and invasion of cells was investigated by cell staining and living bacteria count. The bacterial count results showed that there are differences in the ability of bacteria to adhere and cells at different iron ion concentrations. The number of bacteria that adhere and invade the cells was positively correlated with the iron ion concentration. When the iron ion concentration was between 100 and 400 μM, the ability of the three bacteria strains (ATCC17978, ATCC17978Δ*hcp*, and ATCC17978Δ*hcp^+^*) to adhere and invade cells was significantly different compared to that of the control group. Particularly, the ability of ATCC17978Δ*hcp* to adhere and invade cells was the strongest. The above results indicate that the bacteria are more likely to adhere and invade host cells in the absence of *hcp*. This is consistent with previous reports, which showed that fluctuation of iron content can regulate the expression of outer membrane protein A (OmpA), which acts as an essential component of bacterial adhesion to cells. The change in the OmpA level can mediate the binding of bacteria to host cells (Kentache et al., 2017).

Bacteria are prone to develop environmental susceptibility at a low iron concentration and develop virulence when iron is overloaded (Doherty, 2007; Kang and Kirienko, 2017). This further indicates that the increase in iron ion concentration can promote the number of bacteria that adhere to cells. RTCA results showed that the effects of ATCC17978Δ*hcp* and ATCC17978 on the cell growth index are different. These results suggest that in the *A. baumannii*-cells co-culture, the impact of iron ions on the effect of bacteria in biological conditions differs between ATCC17978 and ATCC17978Δ*hcp*, and this difference is more likely due to the loss of *hcp.* As mentioned earlier, the expression of *hcp* in the ATCC17978-cells co-culture was lower than that in the abiotic environment, suggesting that this behavior may be an escape mechanism from the host cellular immune response (Kang and Kirienko, 2017). Gram staining showed that ATCC17978Δ*hcp* is more likely to adhere and invade human alveolar epithelial cells. Although ATCC17978 and ATCC17978Δ*hcp^+^* bacteria can be found inside the co-cultured cells, most of the bacteria adhered to cells only. Collectively, the above results indicate that ATCC17978Δ*hcp* is more likely to infect cells. Based on previous literature (Doherty, 2007; Burtnick and Brett, 2013; Shyntum et al., 2019) and our experimental results, we speculated that there is a mutual regulatory relationship between T6SS and the iron ion regulation system.

Under the experimental conditions that were used in this study, the *hcp* relative expression level in the *A. baumannii*-cell co-culture was the highest in a concentration of iron ions of 100 μM. It is possible that under this condition, the cells had the highest level of iron ion restriction in the environment, and therefore, bacteria needed to mobilize the related mechanisms, such as secreting siderophores, to obtain iron. Siderophores can influence the host function by modulating cellular iron homeostasis, further providing a mechanism by which resident bacteria influence their local environment at the host–microbial interface (Ellermann and Arthur, 2017).

*A. baumannii* contains up to ten different siderophores that are encoded at three different sites: acinetobactin and pre-acinetobactin, baumannoferrins A and B, and fimsbactins A–F. Acinetobactin is often referred to as the major *A. baumannii* siderophore produced. The expression of multiple siderophores is common in bacterial pathogens and is usually related to virulence (Sheldon and Skaar, 2020).

Although bacterial siderophores have a wide range of biological effects on the pathogen and host (de Pontual, 2017), bacteria can obtain iron ions by secreting siderophores in a low-iron environment; however, the correlation between the secretion of siderophores and T6SS has not been reported. According to our experimental results, we speculate that T6SS promotes the secretion of siderophores and that the release of siderophores enhances the toxicity of bacteria (de Toledo et al., 2018).

However, because this study did not investigate factors related to the iron metabolism system, the exact relationship between T6SS and iron metabolism still needs further studies.

## Data Availability Statement

The datasets presented in this study can be found in online repositories. The names of the repository/repositories and accession number(s) can be found below: NCBI Trace Archive NCBI Sequence Read Archive, accession numbers: OL470900 - OL470903 and OK655938.

## Author Contributions

Conceptualization: DY. Methodology: PP, XW, YC, QC, YY, CW, TC, and DY. Formal analysis: PP, QC, YC, CW, TC, and HW. Investigation: PP, XW, YC, QC, YY, CW, TC, HW, and DY. Writing—original draft: PP, XW, QC, and DY. Writing—review and editing: HW and DY. Project administration: DY. Funding acquisition: DY, HW, and XW. All authors listed have made a substantial, direct, and intellectual contribution to the work and approved it for publication.

## Funding

This work was supported by the National Natural Science Foundation of China (grant number 81930111), the Natural Science Foundation of Zhejiang Province (grant numbers LZ22H190002 and LY17H190001), the Health & Medical Sci-Tech Project of Hangzhou Municipal Health Commission (grant numbers 2018ZD001 and Z2021005), the Science and Technology Project of Hangzhou Municipal (grant number 20191203B91), and the Health & Medical Sci-Tech Project of Health Commission of Zhejiang Province (grant number 2020KY206).

## Conflict of Interest

The authors declare that the research was conducted in the absence of any commercial or financial relationships that could be construed as a potential conflict of interest.

## Publisher’s Note

All claims expressed in this article are solely those of the authors and do not necessarily represent those of their affiliated organizations, or those of the publisher, the editors and the reviewers. Any product that may be evaluated in this article, or claim that may be made by its manufacturer, is not guaranteed or endorsed by the publisher.

## Supplementary Material

Supplementary Figure 1
*hcp* deletion and complementation. The validation of ATCC17978*hcp* knockout and complementation is shown **A–C**. The *hcp* deletion strain and *hcp* complement strain were obtained by the biparental and triparental conjugation methods, respectively. **(A)** The primers *hcp*-up-Mut-F/R, *hcp*-down-Mut-F/R, and *tet*-F/R were used to amplify the gene fragments upstream and downstream of *hcp* and a fragment of the *tet* gene in pKD4, respectively. **(B)** ATCC17978*hcp* gene deletion verification (successful knocked out). **(C)** ATCC17978*hcp* verification of genetic complementation (successful genetic complementation of the target gene).

Supplementary Figure 2 Growth curves for ATCC17978, ATCC17978Δ*hcp*, and ATCC17978Δ*hcp^+^*. The *hcp* mutants of *Acinetobacter baumannii* did not exhibit growth defects.

## References

[B1] Alexandra D. Sophie F. Christophe C. Zimmermann M. B. Christophe L. (2013). Low Iron Availability in Continuous *In Vitro* Colonic Fermentations Induces Strong Dysbiosis of the Child Gut Microbial Consortium and a Decrease in Main Metabolites. FEMS Microbiol. Ecol. 1, 161–175. doi: 10.1111/j.1574-6941.2012.01461.x PMC3511601 22845175

[B2] Aubourg M. Dhalluin A. Gravey F. Pottier M. Giard J. C. (2020). Phenotypic and Proteomic Approaches of the Response to Iron-Limited Condition in Staphylococcus Lugdunensis. BMC Microbiol. 20 (1), 328. doi: 10.1186/s12866-020-02016-x 33115407 PMC7594282

[B3] Balasubramanian R. Rosenzweig A. C. (2008). Copper Methanobactin: A Molecule Whose Time has Come. Curr. Opin. Chem. Biol. 12 (2), 245–249. doi: 10.1016/j.cbpa.2008.01.043 18313412 PMC2685176

[B4] Bethke J. Poblete-Morales M. Irgang R. Yáñez A. Avendaño-Herrera R. (2016). Iron Acquisition and Siderophore Production in the Fish Pathogen Renibacterium Salmoninarum. J. Fish Dis. 39 (11), 1275–1283. doi: 10.1111/jfd.12456 27696458

[B5] Bhargava N. Sharma P. Capalash N. (2010). Quorum Sensing in Acinetobacter: An Emerging Pathogen. Crit. Rev. Microbiol. 36 (4), 349–360. doi: 10.3109/1040841X.2010.512269 20846031

[B6] Bingle L. E. Bailey C. M. Pallen M. J. (2008). Type Vi Secretion: A Beginner's Guide. Curr. Opin. Microbiol. 11 (1), 3–8. doi: 10.1016/j.mib.2008.01.006 18289922

[B7] Braun V. (2001). Iron Uptake Mechanisms and Their Regulation in Pathogenic Bacteria. Int. J. Med. Microbiol. 291 (2), 67–79. doi: 10.1078/1438-4221-00103 11437341

[B8] Burtnick M. N. Brett P. J. (2013). Burkholderia Mallei and Burkholderia Pseudomallei Cluster 1 Type VI Secretion System Gene Expression Is Negatively Regulated by Iron and Zinc. PloS One 8 (10), e76767. doi: 10.1371/journal.pone.0076767 24146925 PMC3795662

[B9] Carruthers M. D. Nicholson P. A. Tracy E. N. Munson R. S. Jr. (2013). Acinetobacter Baumannii Utilizes a Type Vi Secretion System for Bacterial Competition. PloS One 8 (3), e59388. doi: 10.1371/annotation/7aa1688c-56c8-46ca-82ea-f86697f3c4fe 23527179 PMC3602014

[B10] Chen Y. Shao T. Fang S. Pan P. Jiang J. Cheng T.. (2019). Effect of Calcium on the Interaction of Acinetobacter Baumannii With Human Respiratory Epithelial Cells. BMC Microbiol. 19 (1), 264. doi: 10.1186/s12866-019-1643-z 31771504 PMC6880639

[B11] Choi C. H. Lee J. S. Lee Y. C. Park T. I. Lee J. C. (2008). Acinetobacter Baumannii Invades Epithelial Cells and Outer Membrane Protein a Mediates Interactions With Epithelial Cells. BMC Microbiol. 8, 216. doi: 10.1186/1471-2180-8-216 19068136 PMC2615016

[B12] Chow J. Mazmanian S. K. (2010). A Pathobiont of the Microbiota Balances Host Colonization and Intestinal Inflammation. Cell Host Microbe 7 (4), 265–276. doi: 10.1016/j.chom.2010.03.004 20413095 PMC2859213

[B13] Del Pozo J. L. (2018). Biofilm-Related Disease. Expert Rev. Anti Infect. Ther. 16 (1), 51–65. doi: 10.1080/14787210.2018.1417036 29235402

[B14] de Pontual L. (2017). Fer Et Prédisposition Aux Infections [Iron and Susceptibility to Infections]. Arch. Pediatr. Retour Au Numéro 24 (5), 5S14–5S17. doi: 10.1016/S0929-693X(17)24004-4 28622776

[B15] de Toledo L. A. S. Rosseto H. C. Bruschi M. L. (2018). Iron Oxide Magnetic Nanoparticles as Antimicrobials for Therapeutics. Pharm. Dev. Technol. 23 (4), 316–323. doi: 10.1080/10837450.2017.1337793 28565928

[B16] Diana G. Claudio H.-C. Ana R. Pilar G. Patricia R.-M. Christophe B. (2016). Monitoring in Real Time the Formation and Removal of Biofilms From Clinical Related Pathogens Using an Impedance-Based Technology. PloS One 11 (10), e0163966. doi: 10.1371/journal.pone.0163966 27695058 PMC5047529

[B17] Diana G. Lucía F. Beatriz M. Patricia R. M. Pilar G. Ana R. (2017). Real-Time Assessment of Staphylococcus Aureus Biofilm Disruption by Phage-Derived Proteins. Front. Microbiol. 8, 1632. doi: 10.3389/fmicb.2017.01632 28883818 PMC5573737

[B18] Doherty C. P. (2007). Host-Pathogen Interactions: The Role of Iron. J. Nutr. 137 (5), 1341–1344. doi: 10.1093/jn/137.5.1341 17449603

[B19] Ellermann M. Arthur J. C. (2017). Siderophore-Mediated Iron Acquisition and Modulation of Host-Bacterial Interactions. Free Radic. Biol. Med. 105, 68–78. doi: 10.1016/j.freeradbiomed.2016.10.489 27780750 PMC5401654

[B20] Fiester S. E. Nwugo C. C. Penwell W. F. Neary J. M. Beckett A. C. Arivett B. A.. (2015). Role of the Carboxy Terminus of Seca in Iron Acquisition, Protein Translocation, and Virulence of the Bacterial Pathogen Acinetobacter Baumannii. Infect. Immun. 83 (4), 1354–1365. doi: 10.1128/IAI.02925-14 25605767 PMC4363421

[B21] German N. Lüthje F. Hao X. Rønn R. Rensing C. (2016). Microbial Virulence and Interactions With Metals. Prog. Mol. Biol. Trans. Sci. 142, 27–49. doi: 10.1016/bs.pmbts.2016.05.010 27571691

[B22] Grandi G. (2010). Bacterial Surface Proteins and Vaccines. F1000 Biol. Rep. 2, 36. doi: 10.3410/B2-36 20948798 PMC2950030

[B23] Hachani A. Wood T. E. Filloux A. (2016). Type Vi Secretion and Anti-Host Effectors. Curr. Opin. Microbiol. 29, 81–93. doi: 10.1016/j.mib.2015.11.006 26722980

[B24] Hodges K. Hecht G. (2012). Interspecies Communication in the Gut, From Bacterial Delivery to Host-Cell Response. J. Physiol. 590 (3), 433–440. doi: 10.1113/jphysiol.2011.220822 22106176 PMC3379691

[B25] Kang D. Kirienko N. V. (2017). High-Throughput Genetic Screen Reveals That Early Attachment and Biofilm Formation Are Necessary for Full Pyoverdine Production by Pseudomonas Aeruginos. Front. Microbiol. 8, 1707. doi: 10.3389/fmicb.2017.01707 28928729 PMC5591869

[B26] Kehl-Fie T. E. Skaar E. P. (2010). Nutritional Immunity Beyond Iron: A Role for Manganese and Zinc. Curr. Opin. Chem. Biol. 14 (2), 218–224. doi: 10.1016/j.cbpa.2009.11.008 20015678 PMC2847644

[B27] Kentache T. Abdelkrim A. B. Jouenne T. De E. Hardouin J. (2017). Global Dynamic Proteome Study of a Pellicle-Forming Acinetobacter Baumannii Strain. Mol. Cell. Proteomics Mcp 16 (1), 100–112. doi: 10.1074/mcp.M116.061044 27799293 PMC5217776

[B28] Kim J. Lee J.-Y. Lee H. Choi J. Y. Kim D. H. Wi Y. M.. (2017). Microbiological Features and Clinical Impact of the Type VI Secretion System (T6SS) in Acinetobacter Baumannii Isolates Causing Bacteremia. Virulence 8 (7), 1378–1389. doi: 10.1080/21505594.2017.1323164 28448786 PMC5711433

[B29] Kumar A. Alam A. Rani M. Ehtesham N. Z. Hasnain S. E. (2017). Biofilms: Survival and Defense Strategy for Pathogens. Int. J. Med. Microbiol. 307 (8), 481–489. doi: 10.1016/j.ijmm.2017.09.016 28950999

[B30] Lee J. C. Koerten H. van den Broek P. Beekhuizen H. Wolterbeek R. van den Barselaar M.. (2006). Adherence of Acinetobacter Baumannii Strains to Human Bronchial Epithelial Cells. Res. Microbiol. 157 (4), 360–366. doi: 10.1016/j.resmic.2005.09.011 16326077

[B31] Lewis J. M. Deveson Lucas D. Harper M. Boyce J. D. (2019). Systematic Identification and Analysisof Acinetobacter Baumannii Type VISecretion System Effectorand Immunity Components. Front. Microbiol. 10, 2440. doi: 10.3389/fmicb.2019.02440 31736890 PMC6833914

[B32] Lobato-Marquez D. Diaz-Orejas R. Garcia-Del Portillo F. (2016). Toxin-Antitoxins and Bacterial Virulence. FEMS Microbiol. Rev. 40 (5), 592–609. doi: 10.1093/femsre/fuw022 27476076

[B33] Longo F. Vuotto C. Donelli G. (2014). Biofilm Formation in Acinetobacter Baumannii. New Microbiol 37 (2), 119–127. 24858639

[B34] Martins A. C. Almeida J. I. Lima I. S. Kapitão A. S. Gozzelino R. (2017). Iron Metabolism and the Inflammatory Response. IUBMB Life 69 (6), 442–450. doi: 10.1002/iub.1635 28474474

[B35] McQueary C. N. Actis L. A. (2011). Acinetobacter Baumannii Biofilms: Variations Among Strains and Correlations With Other Cell Properties. J. Microbiol. (Seoul Korea) 49 (2), 243–250. doi: 10.1007/s12275-011-0343-7 21538245

[B36] Mihara K. Tanabe T. Yamakawa Y. Funahashi T. Nakao H. Narimatsu S.. (2004). Identification and Transcriptional Organization of a Gene Cluster Involved in Biosynthesis and Transport of Acinetobactin, a Siderophore Produced by Acinetobacter Baumannii ATCC 19606t. Microbiology 150 (Pt 8), 2587–2597. doi: 10.1099/mic.0.27141-0 15289555

[B37] Oh M. H. Lee J. C. Kim J. Choi C. H. Han K. (2015). Simple Method for Markerless Gene Deletion in Multidrug-Resistant Acinetobacter Baumannii. Appl. Environ. Microbiol. 81 (10), 3357–3368. doi: 10.1128/AEM.03975-14 25746991 PMC4407223

[B38] Peneş N. O. Muntean A. A. Moisoiu A. Muntean M. M. Chirca A. Bogdan M. A.. (2017). An Overview of Resistance Profiles Eskape Pathogens From 2010-2015 in a Tertiary Respiratory Center in Romania. Rom. J. Morphol. Embryol. 58 (3), 909–922. 29250670

[B39] Peng K. Qiu Y. Li J. Zhang Z. C. Ji F. H. (2017). Dexmedetomidine Attenuates Hypoxia/Reoxygenation Injury in Primary Neonatal Rat Cardiomyocytes. Exp. Ther. Med. 14 (1), 689–695. doi: 10.3892/etm.2017.4537 28672986 PMC5488536

[B40] Porcheron G. Garénaux A. Proulx J. Sabri M. Dozois C. M. (2013). Iron, Copper, Zinc, and Manganese Transport and Regulation in Pathogenic Enterobacteria: Correlations Between Strains, Site of Infection and the Relative Importance of the Different Metal Transport Systems for Virulence. Front. Cell. Infect. Microbiol. 3, 90. doi: 10.3389/fcimb.2013.00090 24367764 PMC3852070

[B41] Repizo G. D. Espariz M. Seravalle JL and Salcedo S. P. (2019). Bioinformatic Analysis of the Type VISecretion System and Its PotentialToxins in the Acinetobacter Genus. Front. Microbiol. 10, 2519. doi: 10.3389/fmicb.2019.02519 31736933 PMC6838775

[B42] Repizo G. D. Gagné S. Foucault-Grunenwald M.-L. Borges V. Charpentier X. Limansky A. S.. (2015). Differential Role of the T6SS Inacinetobacter Baumannii Virulence. PloS One 10 (9), e0138265. doi: 10.1371/journal.pone.0138265 26401654 PMC4581634

[B43] Runci F. Gentile V. Frangipani E. Rampioni G. Leoni L. Lucidi M.. (2019). Contribution of Active Iron Uptake to Acinetobacter Baumannii Pathogenicity. Infect. Immun. 87 (4), e00755–e00718. doi: 10.1128/IAI.00755-18 30718286 PMC6434119

[B44] Runyen-Janecky L. J. (2013). Role and Regulation of Heme Iron Acquisition in Gram-Negative Pathogens. Front. Cell. Infect. Microbiol. 3, 55. doi: 10.3389/fcimb.2013.00055 24116354 PMC3792355

[B45] Santangelo K. S. Nuovo G. J. Bertone A. L. (2012). *In Vivo* Reduction or Blockade of Interleukin-1β in Primary Osteoarthritis Influences Expression of Mediators Implicated in Pathogenesis. Osteoarthr. Cartil. 20 (12), 1610–1618. doi: 10.1016/j.joca PMC3478416 22935786

[B46] Sha J. Rosenzweig J. A. Kozlova E. V. Wang S. Erova T. E. Kirtley M. L.. (2013). Evaluation of the Roles Played by Hcp and Vgrg Type 6 Secretion System Effectors in Aeromonas Hydrophila Ssu Pathogenesis. Microbiology 159 (Pt6), 1120–1135. doi: 10.1099/mic.0.063495-0 23519162 PMC3709694

[B47] Sheldon J. R. Skaar E. P. (2020). Acinetobacter Baumannii Can Use Multiple Siderophores for Iron Acquisition, But Only Acinetobactin Is Required for Virulence. PloS Pathog. 16 (10), e1008995. doi: 10.1371/journal.ppat.1008995 33075115 PMC7595644

[B48] Shyntum D. Y. Nkomo N. P. Shingange N. L. Gricia A. R. Bellieny-Rabelo D. Moleleki L. N. (2019). The Impact of Type Vi Secretion System, Bacteriocins and Antibiotics on Bacterial Competition of Pectobacterium Carotovorum Subsp. Brasiliense and the Regulation of Carbapenem Biosynthesis by Iron and the Ferric-Uptake Regulator. Front. Microbiol. 10, 2379. doi: 10.3389/fmicb.2019.02379 31681235 PMC6813493

[B49] Singh P. K. Parsek M. R. Greenberg E. P. Welsh M. J. (2002). A Component of Innate Immunity Prevents Bacterial Biofilm Development. Nature 417 (6888), 552–555. doi: 10.1038/417552a 12037568

[B50] Stefanowicz-Hajduk J. Ochocka J. R. (2020). Real-Time Cell Analysis System in Cytotoxicity Applications: Usefulness and Comparison With Tetrazolium Salt Assays. Toxicol. Rep. 7, 335–344. doi: 10.1016/j.toxrep.2020.02.002 32090021 PMC7025972

[B51] Stepanović S. Ćirković I. Ranin L. Svabić-Vlahović M. (2010). Biofilm Formation by Salmonella Spp. And Listeria Monocytogenes on Plastic Surface. Lett. Appl. Microbiol. 38 (5), 428–432. doi: 10.1111/j.1472-765X.2004.01513.x 15059216

[B52] Stoodley P. Sauer K. Davies D. G. Costerton J. W. (2002). Biofilms as Complex Differentiated Communities. Annu. Rev. Microbiol. 56, 187–209. doi: 10.1146/annurev.micro.56.012302.160705 12142477

[B53] Swe Swe-Han K. Mlisana K. P. Pillay M. (2017). Analysis of Clinical and Microbiological Data on Acinetobacter Baumannii Strains Assist the Preauthorization of Antibiotics at the Patient Level for an Effective Antibiotic Stewardship Program. J. Infect. Public Health 10 (5), 608–616. doi: 10.1016/j.jiph.2017.01.014 28237694

[B54] Tang L. Yue S. Li G. Y. Li J. Wang X. R. Li S. F.. (2016). Expression, Secretion and Bactericidal Activity of Type Vi Secretion System in Vibrio Anguillarum. Arch. Microbiol. 198 (8), 751–760. doi: 10.1007/s00203-016-1236-2 27172981

[B55] Teixeira A. B. Barin J. Hermes D. M. Barth A. L. Martins A. F. (2017). PCR Assay Based on the Gyrb Gene for Rapid Identification of Acinetobacter Baumannii-Calcoaceticus Complex at Specie Level. J. Clin. Lab. Anal. 31 (3), e22046. doi: 10.1002/jcla.22046 PMC6816819 27605498

[B56] Tomaras A. P. Dorsey C. W. Edelmann R. E. Actis L. A. (2003). Attachment to and Biofilm Formation on Abiotic Surfaces by Acinetobacter Baumannii: Involvement of a Novel Chaperone-Usher Pili Assembly System. Microbiology 149 (Pt 12), 3473–3484. doi: 10.1099/mic.0.26541-0 14663080

[B57] Tucker A. T. Nowicki E. M. Boll J. M. Knauf G. A. Burdis N. C. Trent M. S.. (2014). Defining Gene-Phenotype Relationships in Acinetobacter Baumannii Through One-Step Chromosomal Gene Inactivation. mBio 5 (4), e01313–e01314. doi: 10.1128/mBio.01313-14 25096877 PMC4128354

[B58] Weaver C. M. Peacock M. (2011). Calcium. Adv. Nutr. 2 (3), 290–292. doi: 10.3945/an.111.000463 22332061 PMC3090164

[B59] Weber B. S. Hennon S. W. Wright M. S. Scott N. E. de Berardinis V. Foster L. J.. (2016). Genetic Dissection of the Type Vi Secretion System in Acinetobacter and Identification of a Novel Peptidoglycan Hydrolase, Tagx, Required for Its Biogenesis. mBio 7 (5), e01253–e01216. doi: 10.1128/mBio.01253-16 27729508 PMC5061870

[B60] Weber B. S. Kinsella R. L. Harding C. M. Feldman M. F. (2017). The Secrets of Acinetobacter Secretion. Trends Microbiol. 25 (7), 532–545. doi: 10.1016/j.tim.2017.01.005 28216293 PMC5474180

[B61] Weber B. S. Ly P. M. Irwin J. N. Pukatzki S. Feldman M. F. (2015). A Multidrug Resistance Plasmid Contains the Molecular Switch for Type VI Secretion in Acinetobacter Baumannii. Proc. Natl. Acad. Sci. U. S. A. 112 (30), 9442–9447. doi: 10.1073/pnas.1502966112 26170289 PMC4522760

[B62] Wong D. Nielsen T. B. Bonomo R. A. Pantapalangkoor P. Spellberg B. (2017). Clinical and Pathophysiological Overview of Acinetobacter Infections: A Century of Challenges. Clin. Microbiol. Rev. 30 (1), 409–447. doi: 10.1128/CMR.00058-16 27974412 PMC5217799

[B63] Wu J. Pugh R. Laughlin R. C. Andrews-Polymenis H. McClelland M. Bäumler A. J.. (2014). High-Throughput Assay to Phenotype Salmonella Enterica Typhimurium Association, Invasion, and Replication in Macrophages. J. Vis. Exp. 90), e51759. doi: 10.3791/51759 PMC4500590 25146526

[B64] Yamamoto S. Okujo N. Sakakibara Y. (1994). Isolation and Structure Elucidation of Acinetobactin, A Novel Siderophore From Acinetobacter Baumannii. Arch. Microbiol. 162 (4), 249–254. doi: 10.1007/BF00301846 7802543

[B65] Zimbler D. L. Penwell W. F. Gaddy J. A. Menke S. M. Tomaras A. P. Connerly P. L.. (2009). Iron Acquisition Functions Expressed by the Human Pathogen Acinetobacter Baumannii. Biometals Int. J. Role Metal Ions Biol. Biochem. Med. 22 (1), 23–32. doi: 10.1007/s10534-008-9202-3 19130255

