# Supplementary figures and images for: Effect of Hcp Iron Ion Regulation on the Interaction Between Acinetobacter baumannii With Human Pulmonary Alveolar Epithelial Cells and Biofilm Formation
Source: Front Cell Infect Microbiol. 2022 Feb 23;12:761604. doi: 10.3389/fcimb.2022.761604 (PMC8905654; doi:10.3389/fcimb.2022.761604)

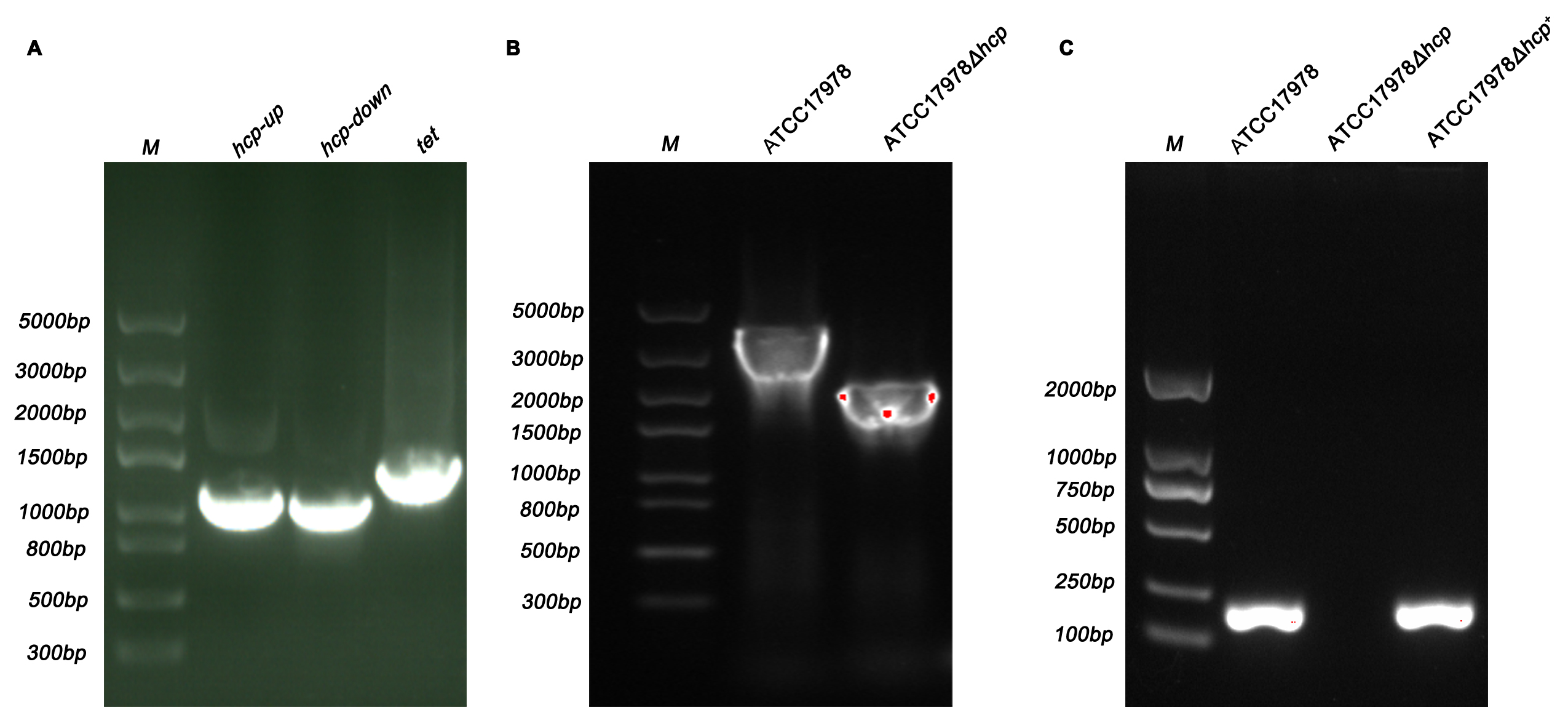

Supplement: Supplementary Figure 1 — hcp deletion and complementation. The validation of ATCC17978hcp knockout and complementation is shown A–C. The hcp deletion strain and hcp complement strain were obtained by the biparental and triparental conjugation methods, respectively. (A) The primers hcp-up-Mut-F/R, hcp-down-Mut-F/R, and tet-F/R were used to amplify the gene fragments upstream and downstream of hcp and a fragment of the tet gene in pKD4, respectively. (B) ATCC17978hcp gene deletion verification (successful knocked out). (C) ATCC17978hcp verification of genetic complementation (successful genetic complementation of the target gene). [file Image_1.jpeg]

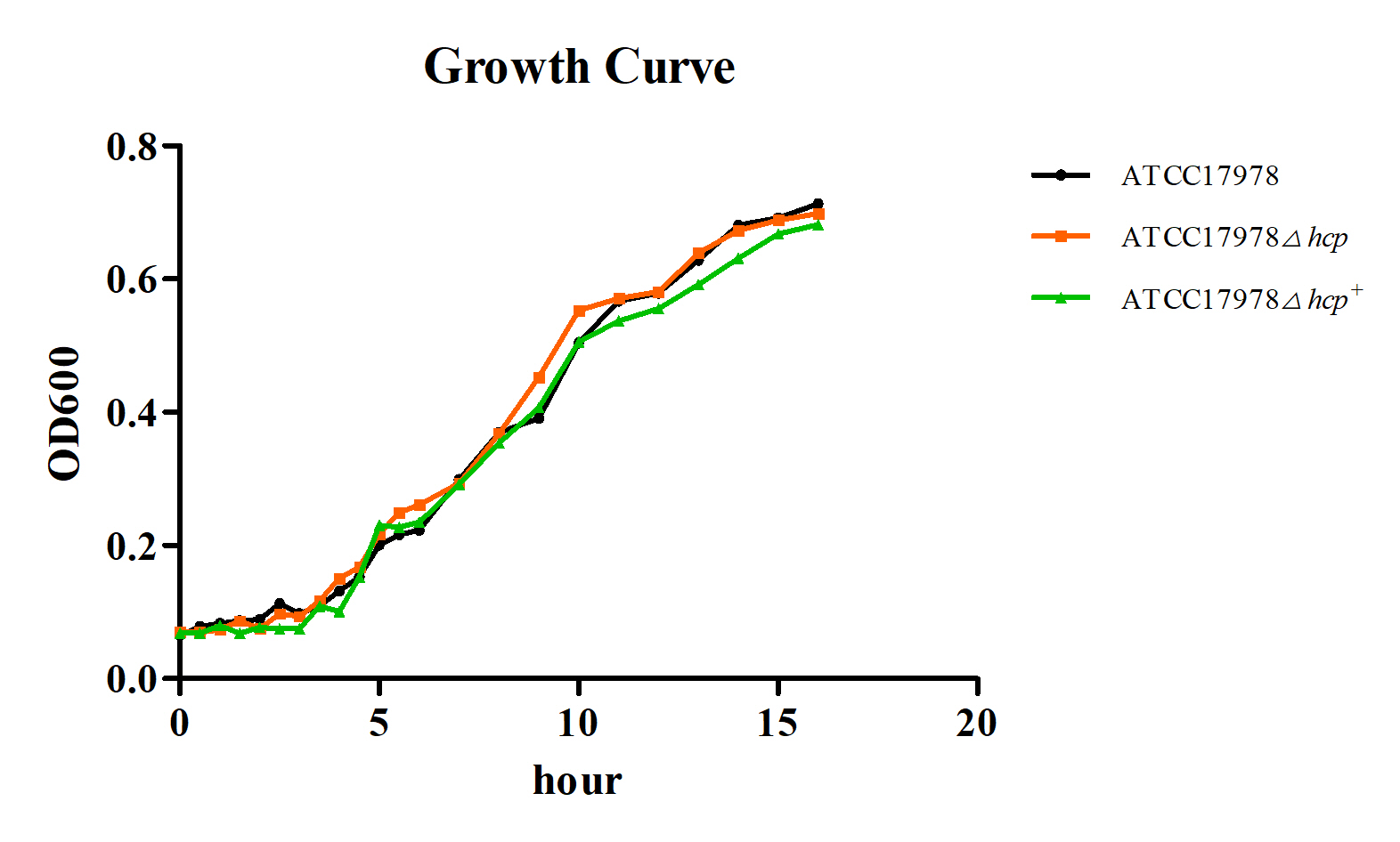

Supplement: Supplementary Figure 2 — Growth curves for ATCC17978, ATCC17978Δhcp, and ATCC17978Δhcp+ . The hcp mutants of Acinetobacter baumannii did not exhibit growth defects. [file Image_2.jpeg]

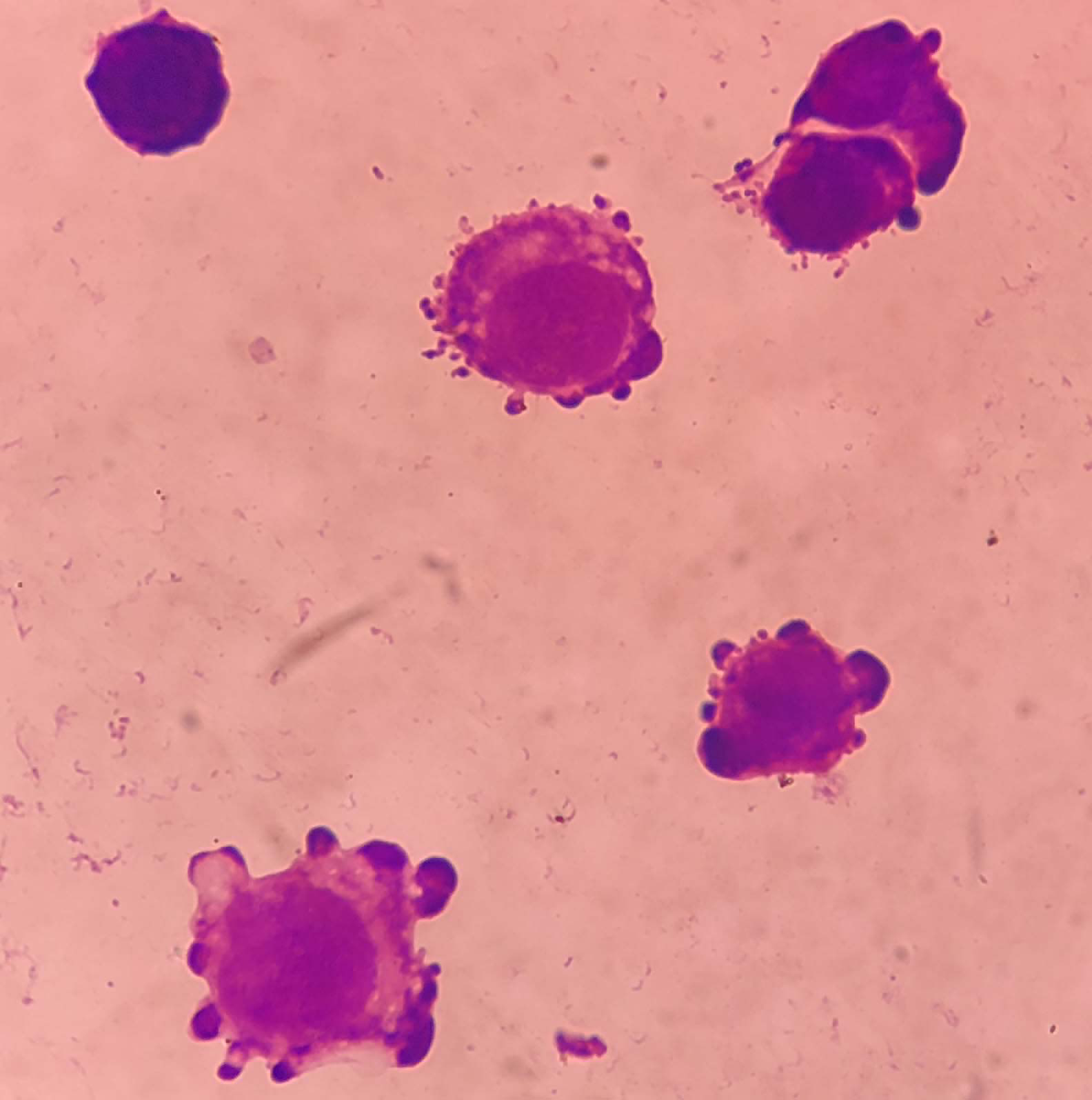

Supplement: Supplementary file 3 [file Image_3.tif]
