# Supplementary material for: Effect of Hcp Iron Ion Regulation on the Interaction Between Acinetobacter baumannii With Human Pulmonary Alveolar Epithelial Cells and Biofilm Formation
Source: Front Cell Infect Microbiol. 2022 Feb 23;12:761604. doi: 10.3389/fcimb.2022.761604 (PMC8905654; doi:10.3389/fcimb.2022.761604)
Supplement: Supplementary file 4 [file Table_1.docx]

**Table S1** Strains, plasmid and primers used in this study

| **Plasmid, strain, or primers** | **Relevant characteristic(s)** | **Reference/Source** |
| --- | --- | --- |
| **Plasmids** | | |
| pDS132 | Suicide vector; *ori*R6K*sacB*; Cmr | ^[29]^ |
| pBR322 | Cloning vector with tetracycline resistance. | ^[30]^ |
| pRK2013 | Combined auxiliary vector for conjugation | ^[12]^ |
| pWH1266 | *E. coli- A. baumannii*shuttle plasmid, Tic^r^, Tet^r^, Amp^r^ | ^[31]^ |
| pMD19-T | General cloning vector | TaKaRa |
| pDS132*::hcp*-*tet* | pDS132 with upstream and downstream of *hcp* and *tet* resistant gene insertion | This study |
| pWH1266*::hcp* | pWH1266 with wild type gene *hcp* | This study |
| pMD19-T*::hcp* | pMD19-T with wild type gene *hcp* | This study |
| pET-RA | *Acinetobacterbaumannii*cloning vector pET-RA with GFP, Rif^r^ | ^[36]^ |
| **Strains** | | |
| *Acinetobacterbaumannii*ATCC17978 | *A.baumannii* prototype strain | This study |
| *E. coli*S17-1 λ pir | General cloning strain, *pir^+^* | ^[29]^ |
| *E. coli*S17-1 λ pir (pDS132*::hcp*-*tet*) | *E. coli*S17-1 λ pir strain with pDS132*::hcp*-*tet*insertion | This study |
| ATCC17978(pDS132*::hcp-tet*) | ATCC17978 with pDS132*::hcp-tet*insertion | This study |
| *E. coli*DH5α | General cloning strain | TaKaRa |
| *E. coli*DH5α (pMD19-T*::hcp*) | *E. coli*DH5α with pMD19-T*::hcp* | This study |
| *E. coli*DH5α (pWH1266) | *E. coli*DH5α with pWH1266 | This study |
| *E. coli*DH5α (pWH1266*::hcp*) | *E. coli*DH5α with pWH1266*::hcp* | This study |
| *E. coli*DH5α (pET-RA) | *E. coli*DH5α with pET-RA | This study |
| ATCC17978Δ*hcp::tet-sacB* | ATCC17978 mutant with *hcp* replaced with *tet-sacB* | This study |
| ATCC17978Δ*hcp* | ATCC 17978 mutant containing a unmarked deletion of *hcp* | This study |
| ATCC17978Δ*hcp*^+^ | ATCC 17978 *hcp* deletion mutant with pWH1266*::hcp* insertion | This study |
| ATCC17978 -GFP | ATCC 17978with pET-RA | This study |
| ATCC17978Δ*hcp*-GFP | ATCC17978Δ*hcp* with pET-RA | This study |
| ATCC17978Δ*hcp*^+^-GFP | ATCC17978Δ*hcp*^+^ with pET-RA | This study |
| **Primers** |  |  |
| *hcp*-up-Mut-F | GGATCGATCCTCTAGAGTCGACGTTGACAGAAGAAGGCGGTAA |  |
| *hcp*-up-Mut-R | ATTACATATTGCTGCGTTGTTGGTTGCGAATAGCAGCACACCAA |  |
| *hcp*-down-Mut-F | AACCAACAACGCAGCAATATG |  |
| *hcp*-down-Mut-R | TGAGAATTCTTGAAGACGAAAGGGCTCACGCACCATATTCATCTCAA |  |
| *tet*-F | GCCCTTTCGTCTTCAAGAATT |  |
| *tet*-R | TTCCCGGGAGAGCTCGATATATTCACAGTTCTCCACAAGA |  |
| pDS132-F | ATATCGAGCTCTCCCGGGAA |  |
| pDS132-R | GTCGACTCTAGAGGATCGATCC |  |
| pDS132-check-F | GAACGGCAGGTATATGTGATG |  |
| pDS132-check-R | GGATGTAACGCACTGAGA |  |
| *hcp*-F | GGGAAGCTTGTTCAGCTGGT |  |
| *hcp-*R | AGTCCACTCAACAGCAGCAT |  |
| pMD19-T-*hcp*-F | AGCTCGGTACCCGGGGATCCACGACTTGTCACTGAGTTACCT |  |
| pMD19-T-*hcp*-R | TTGCATGCCTGCAGGTCGACCTTTATGTCAGCCTCCACCAAA |  |
| pWH1266-*hcp*-F | CACACCCGTCCTGTGGATCCACGACTTGTCACTGAGTTACCT |  |
| pWH1266-*hcp*-R | TCTCAAGGGCATCGGTCGACCTTTATGTCAGCCTCCACCAAA |  |
| pET-RA-F | GAATTAGATGGTGATGTTAATGGG |  |
| pET-RA-R | ATCACTAACTCCCTAGTATCTTTC |  |
