# Supplementary material for: Effect of Hcp Iron Ion Regulation on the Interaction Between Acinetobacter baumannii With Human Pulmonary Alveolar Epithelial Cells and Biofilm Formation
Source: Front Cell Infect Microbiol. 2022 Feb 23;12:761604. doi: 10.3389/fcimb.2022.761604 (PMC8905654; doi:10.3389/fcimb.2022.761604)
Supplement: Supplementary file 5 [file Table_2.docx]

Table S2 Primers and probes for RT-qPCR

| **Primer/probe** | **Sequence** |
| --- | --- |
| Hcp-F | GGGAAGCTTGTTCAGCTGGT |
| Hcp-R | AGTCCACTCAACAGCAGCAT |
| Hcp-P | FAM-CAAATCGACTTCTATCGTGCAAATGGC |
| GyrB-F | TGCGCGCTTTGACAAAATGAT |
| GyrB-R | TCCGGCATTTGACGGAAGAA |
| GyrB-P | VIC-AATTACAGCACTTGGCTGTGGTATTGG |
